# The double burden of anemia and excess weight among schoolchildren in Southern Peru: prevalence, associated factors, and the school food environment

**DOI:** 10.3389/fnut.2026.1965478

**Published:** 2026-10-28

**Authors:** Davis J. Marquez-Guzman, Marianela Pecho-Tataje, Mariela E. Pecho-Tataje, Jose A. Pachas-Atuncar, Luis Curotto-Palomino, Carlos Alberto Dávila-Hernández, M. Adela Valero

**Affiliations:** 1 Departamento de Parasitología, Facultad de Farmacia, Universidad de Valencia, Valencia, Spain; 2 Centro de Investigación Biomédica en Red (CIBER) de Enfermedades Infecciosas, Instituto de Salud Carlos III, Madrid, Spain; 3 Facultad de Enfermería, Universidad Nacional San Luis Gonzaga, Ica, Peru; 4 Facultad de Ciencias Biológicas, Universidad Nacional San Luis Gonzaga, Ica, Peru; 5 Facultad de Medicina Humana, Universidad Nacional San Luis Gonzaga, Ica, Peru

**Keywords:** anemia, double burden of malnutrition, excess weight, middle childhood, Peru

## Abstract

**Background:**

The double burden of malnutrition (DBM) refers to the coexistence of different forms of malnutrition, particularly undernutrition or micronutrient deficiencies alongside overweight or obesity, within individuals, households, or populations. This study aimed to: i) determine the prevalence of the double burden of malnutrition defined as the coexistence of anemia and excess weight, among schoolchildren in southern Peru; ii) investigate factors associated with the double burden of malnutrition, excess weight, and central obesity; and iii) characterize the school food environment.

**Methods:**

A cross-sectional study was conducted among 656 schoolchildren aged 5–13 years from six schools in the province of Ica, Peru. Height-for-age (HAZ), weight-for-age (WAZ), BMI-for-age (BAZ), waist-to-height ratio (WHtR), and hemoglobin concentration were assessed using standardized criteria. The school food environment was evaluated using a structured observational checklist. Multivariable logistic regression models were used to examine factors associated with DBM, excess weight, and central obesity, while a general linear model assessed factors associated with hemoglobin concentration.

**Results:**

Stunting (1.4%), underweight (0.5%) and thinness (1.1%) were uncommon, whereas both excess weight and central obesity (WHtR ≥0.5) affected 50.9% of the schoolchildren. Anemia was identified in 23.5% of participants, and the coexistence of anemia and excess weight (DBM) was observed in 9.9%. After adjustment, anemia was independently associated with lower odds of excess weight, whereas urban residence and older age (11–13 years) were associated with lower odds of central obesity. The school food environment was characterized by the widespread availability of energy-dense, ultra-processed foods and the limited availability of healthier alternatives.

**Conclusion:**

Schoolchildren in the study population exhibited a nutritional profile characterized by the coexistence of anemia and a high burden of excess weight, consistent with nutritional patterns described in populations undergoing the nutrition transition. These findings provide baseline evidence to support integrated school-based nutrition interventions and evidence-based public health policies aimed at addressing multiple forms of malnutrition.

## 1 Introduction

The global landscape of pediatric health is currently marked by a critical epidemiological paradox. Over recent decades, the prevalence of childhood overweight and obesity has escalated substantially worldwide, emerging as a paramount public health challenge (1). Likewise, malnutrition encompassing deficiencies, excesses, or imbalances in energy and/or nutrient intake remains a substantial obstacle to achieving the Sustainable Development Goals (2). According to World Health Organization (WHO) estimates, obesity affects over one billion individuals globally, encompassing 650 million adults, 340 million adolescents, and 39 million children, with an upward trajectory across all age cohorts (3). This metabolic burden entails severe short- and long-term sequelae for physical and psychological health, including depression, heightened susceptibility to infectious diseases, cardiovascular and respiratory disorders, and a compromised risk profile for adult chronic pathologies (4–6). Furthermore, emerging clinical evidence underscores that excess adiposity triggers disturbances in iron homeostasis, accelerating the risk of iron deficiency anemia (7). These findings illustrate that excess adiposity and micronutrient deficiencies, often referred to as “hidden hunger,” are not mutually exclusive; rather, they may coexist within individuals, households, and populations, contributing to the double burden of malnutrition (DBM) (8, 9).

At the individual level, DBM can be operationalized using different combinations of nutritional indicators depending on the population, available indicators, and public health priorities. A systematic review of operational definitions of DBM identified substantial heterogeneity across studies and highlighted the importance of selecting indicators according to the population and research context (10). In the present study, individual-level DBM was specifically defined as the concurrent presence of anemia and excess weight (overweight or obesity according to BMI-for-age) in the same child. Central obesity was evaluated separately as a complementary indicator of adiposity.

Mitigating the DBM through targeted interventions demands a precise understanding of vulnerable populations across the life course, wherein an adequate intake of essential nutrients remains foundational to preventing systemic disease (11). However, equitable access to optimum nutrition is heavily constrained by physical, social, cultural, and economic domains. This disparity disproportionately compromises the physiological well-being of communities subjected to poverty, unemployment, educational marginalization, and ethnic minority status (11).

Within this landscape of socio-economic vulnerability, middle childhood spanning 5 to 13 years of age constitutes a critical, highly transformative developmental bridge between early childhood and adolescence. This school-age cohort undergoes profound, age-specific physiological, somatic, cognitive, and psychosocial shifts, establishing a distinct, nutrition-sensitive window where genetic factors and environmental influences dictate long-term health trajectories (12, 13). Crucially, despite the substantial demographic size of this cohort and its profound public health implications, scientific literature and empirical research regarding the nutritional status and specifically the prevalence of the DBM in these children remain scarce. This critical research gap is significantly amplified in marginalized settings, meaning that vulnerable cohorts within this specific middle childhood range have received markedly less empirical attention than other pediatric age groups (13).

In Peru, the coexistence of different forms of malnutrition represents an emerging public health concern in the context of the ongoing nutritional transition. In the Ica region, official surveillance data indicate a substantial burden of excess weight among the population aged ≥15 years, with overweight and obesity reaching 36.7% and 38.2%, respectively, between 2014 and 2022 (14). However, official regional surveillance data for school-aged children remain limited, particularly for those aged 5–13 years, restricting a comprehensive assessment of the nutritional transition in this population. Within this context, the school food environment is particularly relevant, as foods consumed during the school day may contribute substantially to children's daily dietary intake. The national school feeding program has established nutritional targets whereby school breakfasts provided to beneficiary students contribute approximately 20%−25% of daily energy requirements, with higher contributions when additional meals are provided (15). Furthermore, the Peruvian Ministry of Health established national guidelines for the promotion and protection of healthy eating in public and private basic education institutions through Ministerial Resolution No. 195-2019/MINSA, including measures to promote healthy food environments in school kiosks, cafeterias, and dining facilities. Nevertheless, evidence simultaneously characterizing anemia, excess adiposity, and the school food environment among Peruvian schoolchildren remains scarce.

Therefore, this study aimed to: i) determine the prevalence of the double burden of malnutrition defined as the coexistence of anemia and excess weight, among schoolchildren in southern Peru; ii) investigate factors associated with the double burden of malnutrition, excess weight, and central obesity; and iii) characterize the school food environment.

## 2 Materials and methods

### 2.1 Study population

A cross-sectional study was conducted between April and December 2025 in the province of Ica, located on the southern coast of Peru within the Ica Department (Figure 1). According to official population projections for 2025, the province has an estimated population of 497,565 inhabitants (16). Ica has experienced sustained economic growth driven by agriculture and agro-export activities, accompanied by substantial internal and external migration (17). These demographic and socioeconomic changes have contributed to a context of nutritional transition, where urban and rural populations coexist, making the province a relevant setting for investigating nutritional disparities and the double burden of malnutrition among school-aged children.

**Figure 1:**
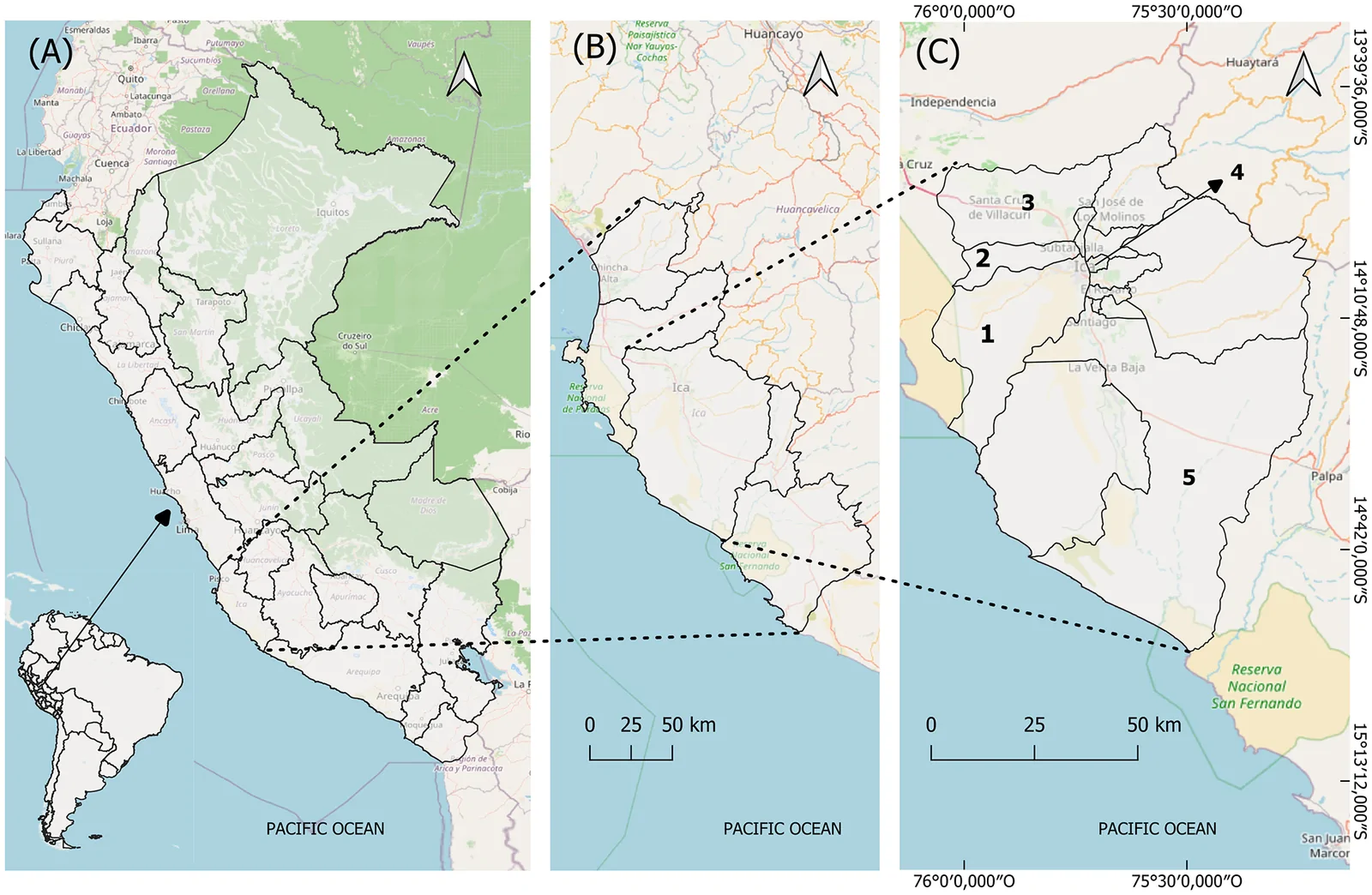
Geographic location of the study area in southern Peru. (A) Location of Peru. (B) Ica Department. (C) Ica Province showing the five participating districts: (1) Ica, (2) Subtanjalla, (3) Salas, (4) Tate, and (5) Santiago. The figure was created in QGIS using vector data from OpenStreetMap (https://www.openstreetmap.org), and administrative boundaries obtained from GADM (https://gadm.org, free for academic use). All data were processed in QGIS. The final figure is published under the Creative Commons Attribution 4.0 (CC BY 4.0) license.

Six educational institutions were selected from five districts representing both urban and rural settings: Ica (urban), Subtanjalla (predominantly urban), Salas (predominantly rural), and Tate and Santiago, both characterized by extensive agricultural areas (Figure 1). Schools were selected by convenience based on their willingness to participate and to ensure representation of both urban and rural settings within the province. A total of 656 schoolchildren aged 5–13 years were included in the study, comprising 411 children from rural areas and 245 from urban areas. Urban–rural classification was determined considering the geographic location of each school and the official classification of each educational institution as urban or rural by the Peruvian Ministry of Education (MINEDU), as reported in the official consolidated registry of public educational institutions (Ministerial Resolution No. 71-2015-MINEDU).

### 2.2 Data collection

#### 2.2.1 Nutritional assessment

Anthropometric measurements were performed by trained nursing staff following standardized procedures. Body weight was measured using calibrated digital scales while participants were barefoot and wearing light clothing. Height was assessed using a portable stadiometer with participants standing upright and their heads positioned in the Frankfurt horizontal plane. Waist circumference (WC) was measured using a non-elastic measuring tape at the midpoint between the lower costal margin and the iliac crest. Measurements were conducted at the participating schools in designated indoor areas that provided adequate privacy and sufficient space for anthropometric assessment. The same measurement procedures and equipment were used across all participating schools to ensure consistency.

Height-for-age (HAZ), weight-for-age (WAZ), and BMI-for-age z-scores (BAZ) were calculated according to the WHO growth reference standards (18). Stunting was defined as HAZ <−2 standard deviations (SD), whereas underweight was defined as WAZ <−2 SD. Based on BMI-for-age, thinness was defined as BAZ <−2 SD, normal nutritional status as BAZ ≥ −2 SD and ≤ +1 SD, overweight as BAZ > +1 SD and <+2 SD, and obesity as BAZ ≥ +2 SD. WAZ scores were calculated only for schoolchildren younger than 10 years of age (18).

The waist-to-height ratio (WHtR) was calculated by dividing waist circumference by height using measurements expressed in the same units. A WHtR ≥ 0.5 was considered indicative of central obesity, a threshold widely recognized as a simple and effective indicator of increased cardiometabolic risk in children and adolescents (19). Central obesity was evaluated as a complementary indicator of adiposity and was not included in the operational definition of DBM.

Hemoglobin concentration was measured using a portable spectrophotometer (HemoCue Hb 301+, HemoCue AB, Ängelholm, Sweden) with a precision of ±0.3 g/dL. Anemia was defined according to WHO criteria as hemoglobin concentrations <11.5 g/dL in children aged 5–11 years and <12.0 g/dL in those aged 12–14 years (20).

For the purposes of this study, excess weight was defined as the presence of overweight or obesity according to BMI-for-age. Individual-level double burden of malnutrition (DBM) was operationally defined as the concurrent presence of anemia and excess weight. Based on anemia and excess weight status, participants were classified into four mutually exclusive nutritional profiles: no anemia and no excess weight (NA/NEW), anemia only (AN), excess weight only (EW), and double burden of malnutrition (DBM). These categories were used to describe the distribution of nutritional profiles according to area of residence.

#### 2.2.2 Household-level variables

Household-level variables comprised place of residence (urban or rural), housing characteristics (cement flooring and electricity), and access to basic household services (improved water supply and sanitation). Information on these variables was obtained through a structured questionnaire completed by the children's parents or legal guardians.

#### 2.2.3 School food environment assessment

The school food environment was assessed through direct observation using selected items from Section C (“Compliance with the Healthy Eating Law”) of the official Monitoring and Evaluation Form for School Kiosks in Public and Private Regular Basic Education Institutions, within the framework of Ministerial Resolution No. 195-2019/MINSA. Only items relevant to food availability and the promotion of healthy eating were considered; therefore, the overall classification of school kiosks as healthy, in process, or unhealthy was not applied. During each school visit, the availability of foods and beverages and other food-environment characteristics was recorded using the criteria established in the official form. For descriptive analysis and graphical presentation, observed food items were grouped into standardized categories according to their characteristics (e.g., sugar-sweetened beverages, confectionery, packaged cookies, and fried foods), while foods meeting the criteria specified in Annex 1 of Ministerial Resolution No. 195-2019/MINSA were considered healthy options. Availability was defined as the observed presence of each category in the school kiosk at the time of assessment and was recorded dichotomously for each school (present/absent). Results were summarized as the number and percentage of schools in which each category or food-environment characteristic was observed.

### 2.3 Statistical analysis

Anthropometric indices (HAZ, WAZ, and BAZ) were calculated using WHO AnthroPlus software (World Health Organization, Geneva, Switzerland). Descriptive statistics included absolute and relative frequencies for categorical variables and means with standard deviations (SD) for continuous variables. Associations between categorical variables were assessed using Pearson's chi-square test.

Demographic variables (age, sex, and place of residence) were included *a priori* in the multivariable models based on their epidemiological relevance, regardless of their statistical significance in the univariable analysis. Household-level variables were considered for inclusion in the multivariable models when they showed an association at *p* < 0.20 in the univariable analysis. This less restrictive threshold was used as a screening criterion to avoid excluding potentially relevant household-level factors that might not reach conventional statistical significance in univariable analyses. Multivariable logistic regression models were fitted to identify factors independently associated with DBM, excess weight, and central obesity. For the DBM model, anemia and excess weight were not evaluated as predictors because both conditions constitute the operational definition of DBM. Adjusted odds ratios (aORs) and their corresponding 95% confidence intervals (95% CIs) were estimated. To assess the potential effect of clustering of participants within schools, sensitivity analyses were conducted using generalized estimating equations (GEE) with school as the clustering variable, a binomial distribution with logit link, and an exchangeable working correlation structure. Given the small number of school clusters (*n* = 6), the Mancl–DeRouen small-sample correction was applied to the sandwich variance estimator. These analyses were performed in R and are reported in Supplementary Table S1.

A general linear model (GLM) was fitted to evaluate the effects of age group, sex, and place of residence on hemoglobin concentration. Main effects and interaction terms were assessed, and F statistics with corresponding *p*-values were reported. Statistical analyses were performed using IBM SPSS Statistics version 28.0.1.1 (IBM Corp., Armonk, NY, USA). Figures were generated using GraphPad Prism version 9.5.1 (GraphPad Software, San Diego, CA, USA) and R software version 4.4.1 (R Foundation for Statistical Computing, Vienna, Austria). Statistical significance was established at *p* < 0.05.

### 2.4 Ethics statement

This study was approved by the Research Ethics Committee of the National University San Luis Gonzaga, Ica, Peru (Certificate No. CEI-UNICA-N041/09-2025; March 20, 2025). Authorization was obtained from the participating educational institutions prior to data collection. As the study population consisted of minors, written informed consent was obtained from parents or legal guardians before enrollment. Participation was voluntary, and the confidentiality and anonymity of all collected information were strictly maintained throughout the study. Anthropometric and hemoglobin assessment results were communicated to the corresponding educational authorities and parents when necessary, and children identified with anemia or nutritional disorders were referred to local health services for appropriate evaluation and follow-up.

## 3 Results

The sociodemographic characteristics of the study population are presented in Table 1. A total of 656 schoolchildren were included, with a mean age of 8.9 ± 1.8 years. Children aged 8–10 years represented the largest age group (49.7%).

**Table 1:** Characteristics of the study population.

Study population	Rural	Urban	Total
	*n* (%)	*n* (%)	*n* (%)
Male sex	175 (42.6)	105 (42.9)	280 (42.7)
Age (years)			
5–7	135 (32.8)	48 (19.6)	183 (27.9)
8–10	208 (50.6)	118 (48.2)	326 (49.7)
11–13	68 (16.5)	79 (32.2)	147 (22.4)
Age mean ± SD	8.6 ± 1.8	9.5 ± 1.8	8.9 ± 1.8

SD, standard deviation.

The nutritional characteristics of the study population are summarized in Table 2. Stunting and underweight were uncommon, affecting 1.4% (9/656) and 0.5% (3/656) of participants, respectively. Based on BMI-for-age, 26.2% (172/656) were classified as overweight and 24.7% (162/656) as obese. Overall, 50.9% (334/656) of participants presented excess weight (overweight or obesity). Based on the waist-to-height ratio (WHtR), 50.9% (334/656) had central obesity, with prevalences of 55.0% and 44.1% among rural and urban participants, respectively. The overall prevalence of anemia was 23.5% (154/656), indicating that nearly one in four children had hemoglobin concentrations below the WHO age-specific cut-off values. Overall, the coexistence of anemia and excess weight, defined as the double burden of malnutrition (DBM), was observed in 9.9% (65/656) of participants.

**Table 2:** Nutritional characteristics of schoolchildren.

Nutritional status	Rural	Urban	Total
	*n* (%)	*n* (%)	*n* (%)
Height-for-age (HAZ)			
Stunted	7 (1.7)	2 (0.8)	9 (1.4)
Normal	404 (98.3)	243 (99.2)	647 (98.6)
Weight-for-Age (WAZ)^*^			
Underweight	0 (0.0)	3 (1.2)	3 (0.5)
Normal	411 (100.0)	242 (98.8)	653 (99.5)
BMI-for-Age (BAZ)			
Thinness	3 (0.7)	4 (1.6)	7 (1.1)
Normal	206 (50.1)	109 (44.5)	315 (48.0)
Overweight	102 (24.8)	70 (28.6)	172 (26.2)
Obesity	100 (24.3)	62 (25.3)	162 (24.7)
Waist-to-height ratio (WHtR)			
WHtR <0.5	185 (45.0)	137 (55.9)	322 (49.1)
WHtR ≥ 0.5	226 (55.0)	108 (44.1)	334 (50.9)
Hemoglobin status (HB)			
Hb mean ± SD	12.3 ± 1.1	12.5 ± 1.0	12.3 ± 1.1
*V*_min._-*V*_max_	7.9–16.2	10.0–15.7	7.9–16.2
Anemia	107 (26.0)	47 (19.2)	154 (23.5)

^*^Calculated only for participants <10 years (*n* = 509).

The household and environmental characteristics of the study population are summarized in Table 3. Most households had access to improved water supply (81.9%), electricity (96.2%), cement flooring (89.9%), and improved sanitation facilities (90.5%). Compared with rural households, urban households had greater access to improved water supply and improved sanitation facilities.

**Table 3:** Household living conditions of the study population.

Household characteristics	Rural	Urban	Total
	*n* (%)	*n* (%)	*n* (%)
Improved water supply	313 (76.2)	224 (91.4)	537 (81.9)
Electricity	393 (95.6)	238 (97.1)	631 (96.2)
Cement floor	363 (88.3)	227 (92.7)	590 (89.9)
Improved sanitation	361 (87.8)	233 (95.1)	594 (90.5)

The distribution of nutritional profiles by area of residence is shown in Figure 2. Although the prevalence of DBM was higher among rural than urban schoolchildren (11.4% vs. 7.3%), the difference was not statistically significant (χ^2^ = 2.87, *p* = 0.090). Similarly, the prevalence of excess weight did not differ significantly between rural and urban areas (49.1% vs. 53.9%; χ^2^ = 1.37, *p* = 0.241). In contrast, central obesity was significantly more prevalent among rural than urban schoolchildren (55.0% vs. 44.1%; χ^2^ = 7.31, p = 0.007). Anemia was also significantly more prevalent among rural than urban schoolchildren (26.0% vs. 19.2%; χ^2^ = 4.01, *p* = 0.045).

**Figure 2:**
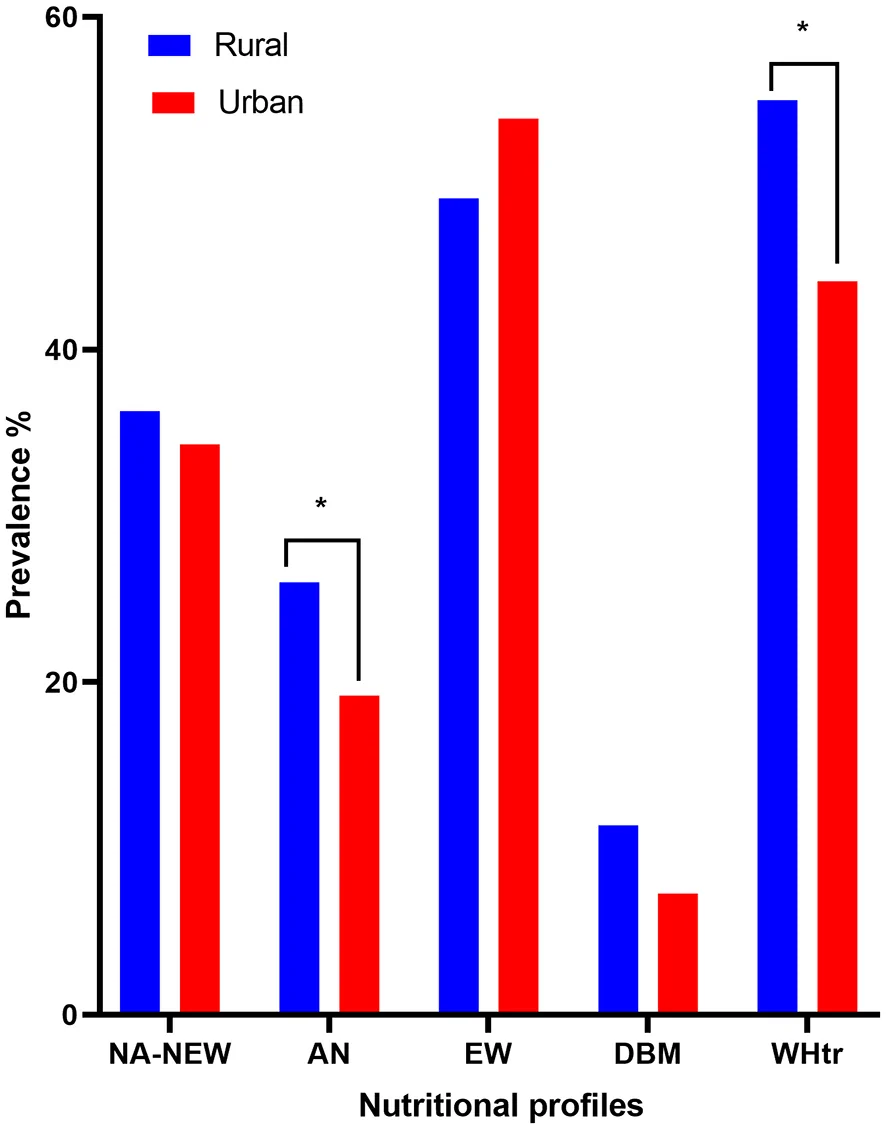
Nutritional profiles by area of residence. NA/NEW, no anemia and no excess weight; **AN**, anemia only; **EW**, excess weight only; **DBM**, double burden of malnutrition (coexistence of anemia and excess weight); WHtR, waist-to-height ratio (≥ 0.5). *p < 0.05 for comparisons between rural and urban schoolchildren using Pearson's chi-square test.

Figure 3 shows the age-specific prevalence of anemia, excess weight, and the double burden of malnutrition (DBM). The prevalence of anemia remained relatively constant across age groups, with no consistent differences between boys and girls. In contrast, excess weight affected approximately half of the children and remained consistently high between 6 and 12 years of age. The prevalence of DBM also showed only modest variation across age groups, with no evident age- or sex-specific pattern. The fluctuations observed at the youngest and oldest ages should be interpreted with caution owing to the limited number of participants in these age groups.

**Figure 3:**
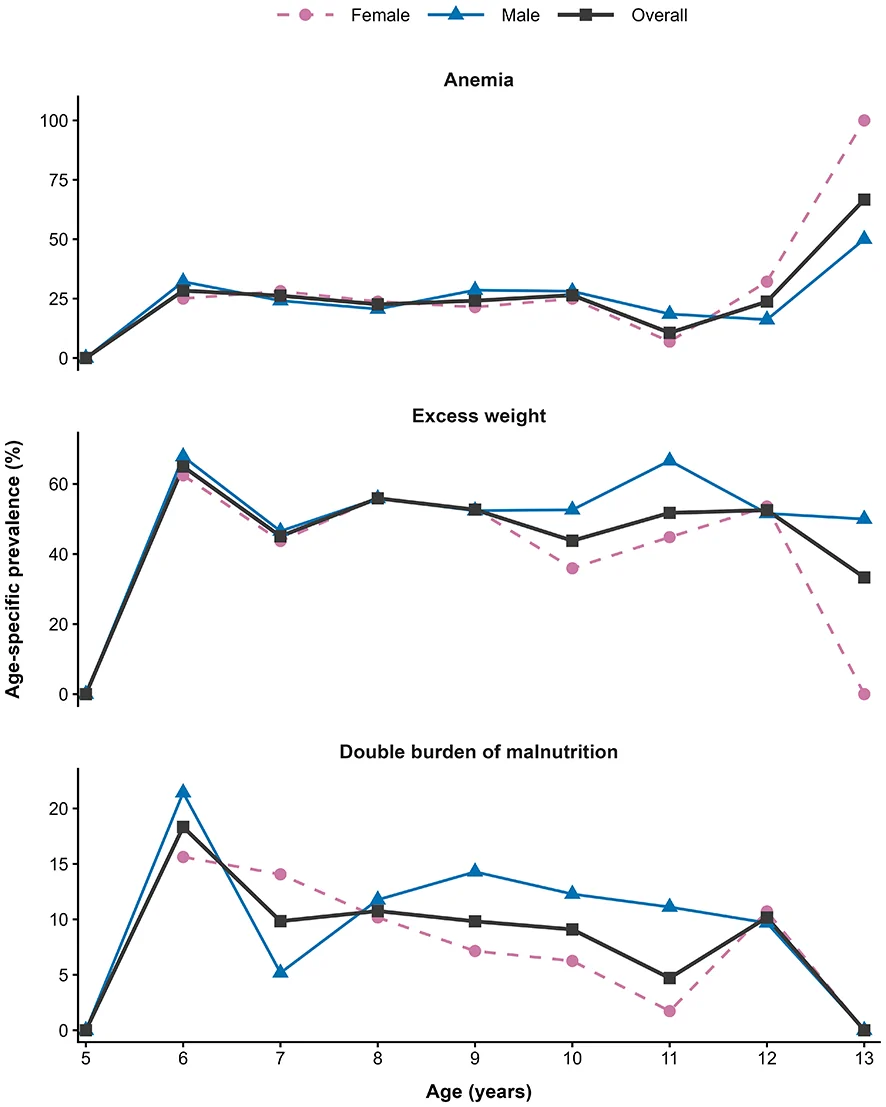
Age-specific distribution of anemia, excess weight, and the double burden of malnutrition.

No statistically significant associations were observed between the demographic or household characteristics evaluated and DBM in the univariable analyses (Table 4). In the adjusted model, sex, age group, and area of residence were not significantly associated with DBM (all *p* > 0.05).

**Table 4:** Factors associated with the double burden of malnutrition among schoolchildren in Ica, Peru.

Variables	Univariate			Multivariate		
	OR	95% CI	*p*-value	aOR	95% CI	*p*-value
Sex						
Female^†^						
Male	1.34	[0.80–2.24]	0.262	1.32	[0.79–2.21]	0.296
Age (Years)						
5–7^†^						
8–10	0.76	[0.43–1.34]	0.338	0.80	[0.45–1.43]	0.457
11–13	0.51	[0.23–1.10]	0.087	0.58	[0.26–1.27]	0.173
Place of residence						
Rural^†^						
Urban	0.61	[0.35–1.08]	0.092	0.66	[0.37–1.18]	0.165
Cement floor	0.92	[0.40–2.11]	0.842			
Non-piped water supply	1.10	[0.56–2.17]	0.789			
Non-flush sanitation	1.03	[0.43–2.49]	0.949			
No electricity	2.71	[0.36–20.4]	0.333			

^†^Indicates the reference category; Demographic variables (age, sex, and area of residence) were included a priori in the multivariable model. Household-level variables were considered for inclusion when *p* < 0.20 in univariable analysis; none met this criterion. Anemia and excess weight were not evaluated as predictors because they constitute the components used to define DBM.

The results of the logistic regression analysis for excess weight are presented in Table 5 and summarized in Figure 4A. In the univariate analysis, schoolchildren with anemia had lower odds of excess weight than those without anemia (OR = 0.63, 95% CI: 0.44–0.91, *p* = 0.014). This association remained significant after adjustment for sociodemographic and household characteristics (aOR = 0.63, 95% CI: 0.43–0.91, *p* = 0.014). Among household characteristics, the absence of flush sanitation was independently associated with higher odds of excess weight (aOR = 2.01, 95% CI: 1.11–3.62, *p* = 0.021). Although non-piped water supply was associated with excess weight in the univariate analysis (OR = 1.55, 95% CI: 1.04–2.31, *p* = 0.033), this association was no longer statistically significant after adjustment. No significant associations were observed for age, sex, place of residence, or cement floor.

**Table 5:** Factors associated with excess weight among schoolchildren in Ica, Peru.

Variables	Univariate			Multivariate		
	OR	95% CI	*p*-value	aOR	95% CI	*p*-value
Sex						
Female^†^						
Male	1.26	[0.93–1.73]	0.136	1.30	[0.94–1.78]	0.110
Age (Years)						
5–7^†^						
8–10	0.96	[0.67–1.38]	0.819	0.88	[0.61–1.28]	0.506
11–13	1.01	[0.66–1.57]	0.952	0.87	[0.55–1.37]	0.538
Place of residence						
Rural^†^						
Urban	0.83	[0.60–1.13]	0.241	1.07	[0.77–1.50]	0.679
Anemia	0.63	[0.44–0.91]	0.014^*^	0.63	[0.43–0.91]	0.014^*^
Cement floor	1.68	[0.99–2.82]	0.050	1.41	[0.81–2.44]	0.222
Non-piped water supply	1.55	[1.04–2.31]	0.033^*^	1.36	[0.89–2.08]	0.153
Non-flush sanitation	2.36	[1.35–4.11]	0.003^*^	2.01	[1.11–3.62]	0.021^*^
No electricity	0.96	[0.43–2.13]	0.912			

^†^Indicates the reference category; ^*^p-value <0.05. Variables included in the adjusted model were selected as described in the Statistical Analysis section.

**Figure 4:**
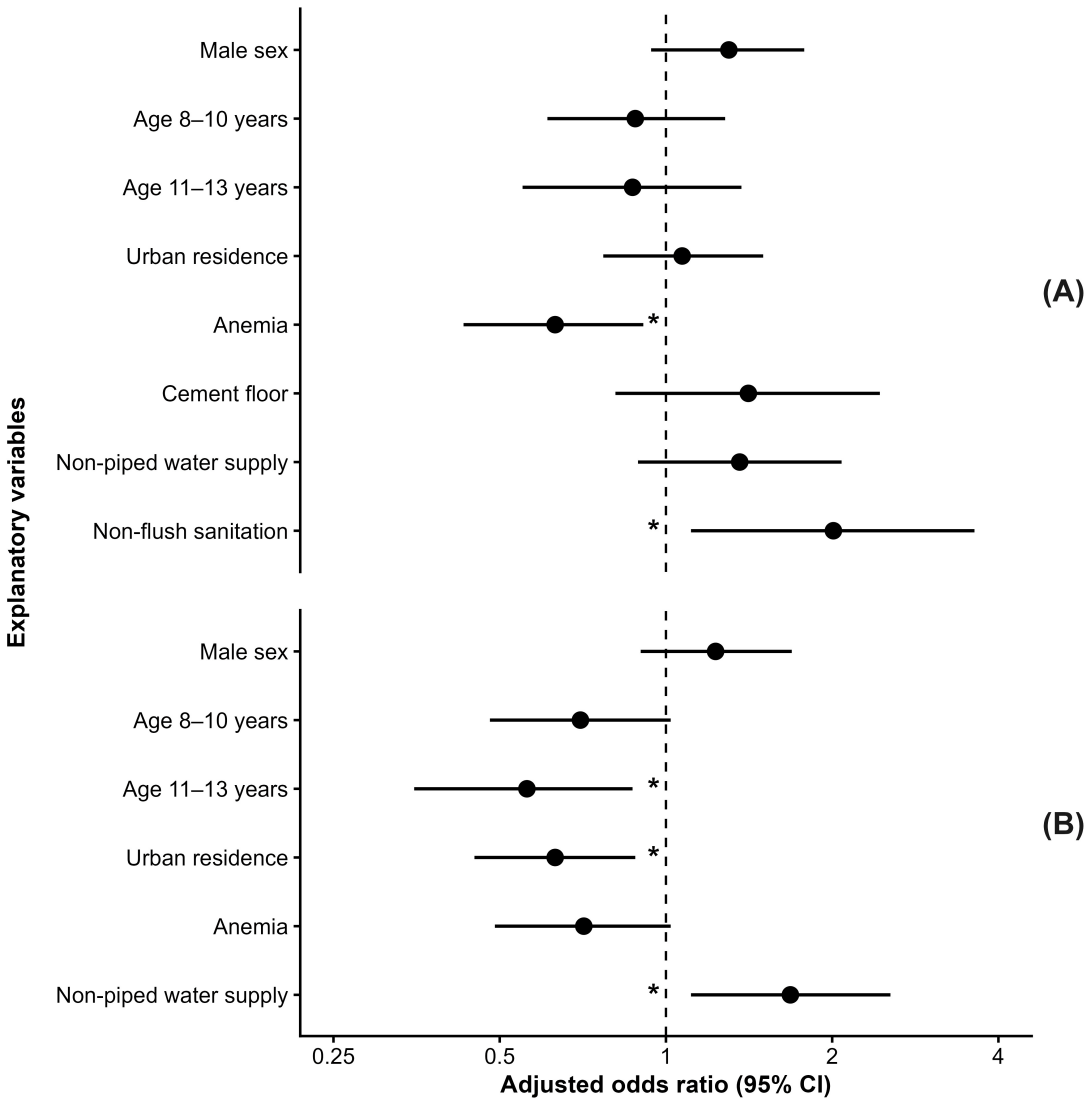
Multivariable analysis of factors associated with nutritional outcomes. (A) Excess weight; (B) Central obesity.

The results of the logistic regression analysis for central obesity are presented in Table 6 and summarized in Figure 4B. In the multivariable analysis, schoolchildren living in urban areas had lower odds of central obesity than those living in rural areas (aOR = 0.63, 95% CI: 0.45–0.88, *p* = 0.006). Likewise, children aged 11–13 years were less likely to present central obesity than those aged 5–7 years (aOR = 0.56, 95% CI: 0.35–0.87, *p* = 0.011). In contrast, living in households without piped water was independently associated with higher odds of central obesity (aOR = 1.68, 95% CI: 1.11–2.55, *p* = 0.014). Although anemia showed an inverse association with central obesity after adjustment, this association did not reach statistical significance (aOR = 0.71, 95% CI: 0.49–1.02, *p* = 0.066). No significant associations were observed for sex. In sensitivity analyses accounting for clustering by school, adjusted effect estimates were highly similar to those obtained from the primary logistic regression models, although confidence intervals and statistical significance varied for some covariates. Estimated within-school correlations were small (α = 0.018 for DBM, −0.006 for excess weight, and −0.002 for central obesity; Supplementary Table S1).

**Table 6:** Factors associated with central obesity among schoolchildren in Ica, Peru.

Variables	Univariate			Multivariate		
	OR	95% CI	*p*-value	Aor	95% CI	*p*-value
Sex						
Female^†^						
Male	1.24	[0.91–1.68]	0.183	1.23	[0.90 – 1.69]	0.201
Age (Years)						
5–7^†^						
8–10	0.69	[0.48–1.00]	0.051	0.70	[0.48–1.02]	0.061
11–13	0.52	[0.34–0.81]	0.004^*^	0.56	[0.35–0.87]	0.011^*^
Place of residence						
Rural^†^						
Urban	0.65	[0.46–0.89]	0.007^*^	0.63	[0.45–0.88]	0.006^*^
Anemia	0.78	[0.54–1.12]	0.173	0.71	[0.49–1.02]	0.066
Cement floor	1.04	[0.63–1.73]	0.88			
Non-piped water supply	1.43	[0.96–2.13]	0.083	1.68	[1.11–2.55]	0.014^*^
Non-flush sanitation	0.97	[0.57–1.64]	0.908			
No electricity	0.96	[0.43–2.13]	0.912			

^†^Indicates the reference category; ^*^ *p*-value <0.05. Variables included in the adjusted model were selected as described in the Statistical Analysis section.

In a general linear model including age group, sex, and place of residence, hemoglobin concentration differed significantly according to age group (F = 10.82, *p* < 0.001) and place of residence (F = 4.51, *p* = 0.034), but not according to sex (F = 0.003, *p* = 0.955). None of the two-way or three-way interactions among the study factors were statistically significant (all *p* > 0.05).

The school food environment was characterized by the widespread availability of sugar-sweetened beverages, fried foods, confectionery, and industrially packaged cookies, whereas healthier alternatives such as fruits, vegetables, and healthy prepared foods were less frequently available (Figure 5). No school displayed food advertising, healthy eating promotional materials, or evidence of restrictions on the sale of unhealthy food products.

**Figure 5:**
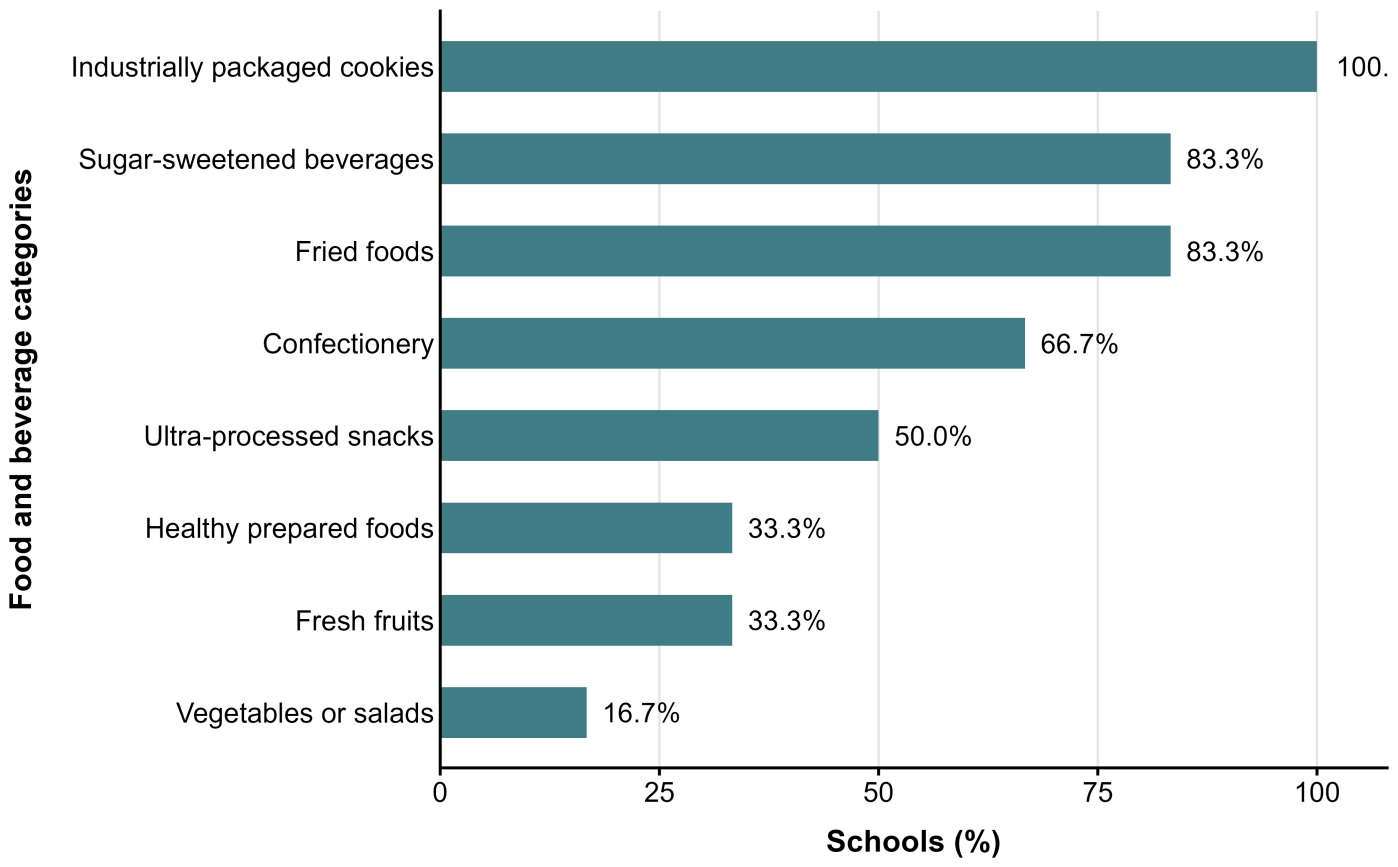
Availability of healthy and unhealthy foods and beverages in participating schools. Values represent the percentage of assessed schools in which each food or beverage category was observed, calculated based on the number of schools where the category was available relative to the total number of schools assessed (*n* = 6). Observed food and beverage items were grouped into standardized categories to facilitate interpretation and graphical presentation.

## 4 Discussion

Previous epidemiological studies conducted in Peru have focused primarily on children under five years of age or on mother–child pairs, assessing the DBM through the coexistence of stunting and overweight or obesity or through household-level forms of malnutrition (21–24). Nevertheless, no previous study has evaluated the coexistence of anemia and excess weight among Peruvian school-aged children, despite this being a critical stage for growth, cognitive development, and the establishment of lifelong dietary and lifestyle behaviors.

The present study provides new evidence on the nutritional profile of Peruvian schoolchildren aged 5–13 years. While chronic undernutrition was uncommon, excess weight affected approximately half of the study population and anemia remained a moderate public health problem. Notably, nearly one in ten schoolchildren presented the coexistence of anemia and excess weight, highlighting the presence of the double burden of malnutrition during the school years.

The low prevalence of stunting, underweight, and thinness indicates that traditional forms of undernutrition were uncommon in the study population, whereas excess weight was highly prevalent. This nutritional profile is consistent with patterns described in the context of the nutritional transition in many low- and middle-income countries, where undernutrition and micronutrient deficiencies coexist with a high burden of excess adiposity (25).

Despite the low prevalence of chronic undernutrition observed in the study population, anemia remained a moderate public health problem according to the WHO classification (20). The coexistence of anemia with a high prevalence of excess weight illustrates that excess adiposity does not necessarily imply adequate micronutrient status, allowing different forms of malnutrition to coexist within the same population.

### 4.1 The nutrition transition and childhood obesity

Evidence from Peru indicates a substantial decline in chronic undernutrition among children under five years of age over the past two decades, reflecting significant improvements in child nutritional status (21–23). This remarkable reduction has been attributed to the expansion of antipoverty policies and improvements in access to health care implemented since the mid-2000s (26, 27). Previous evidence from the province of Ica documented a stunting prevalence of 9.3% among children under five years of age, alongside a marked national decline in chronic undernutrition between 2010 and 2016 (28). In the present study, chronic undernutrition was uncommon among school-aged children. Although direct comparisons with studies conducted in younger age groups should be interpreted cautiously, this finding indicates a low burden of traditional forms of undernutrition in the study population.

In contrast, excess weight affected approximately one in two schoolchildren in our study, a prevalence substantially higher than those reported in previous studies from Latin America. A systematic review estimated a pooled childhood obesity prevalence of 12.2% in Brazil (29), whereas a multicountry analysis reported prevalences ranging from 3.0% to 27.1%, including 26.0% in Ecuador and 18.3% in Bolivia (30). Likewise, studies conducted among children under five years of age reported markedly lower prevalences, including 5.5% in Ecuador (31) and 1.5% in Peru (32). Although direct comparisons should be interpreted cautiously because of differences in study populations and age groups, these comparisons highlight the substantial burden of excess weight observed among the school-aged children included in the present study.

The high prevalence of excess weight observed in our study is consistent with nutritional patterns reported in populations undergoing the nutrition transition in many low- and middle-income countries. This transition, driven by urbanization, economic development, changing family dynamics, greater availability of ultra-processed foods, and increasingly sedentary lifestyles, has contributed to the rapid rise in childhood overweight and obesity worldwide (25, 33).

This finding is of particular concern because childhood obesity is associated with an increased likelihood of persisting into adolescence and adulthood, where it substantially increases the risk of type 2 diabetes, hypertension, metabolic syndrome, non-alcoholic fatty liver disease, and cardiovascular disease (34, 35). Consistent evidence from meta-analyses has shown that elevated body mass index during childhood is associated with a higher risk of cardiometabolic morbidity later in life (36).

### 4.2 Determinants of excess weight and central obesity

The rapid increase in childhood obesity has become a major public health concern in Peru. National analyses have shown that the prevalence of obesity increased across all socioeconomic groups between 1992 and 2017, indicating that the obesity epidemic is no longer restricted to specific population subgroups but has become a widespread phenomenon (22). Consistent with this national trend, our multivariable analyses identified several factors independently associated with excess weight and central obesity among school-aged children.

In the primary multivariable analyses, anemia and non-flush sanitation were associated with excess weight, whereas urban residence, older age (11–13 years), and non-piped water supply were associated with central obesity. Sensitivity analyses accounting for within-school clustering yielded generally similar effect estimates; however, statistical significance varied for some covariates. The association between anemia and excess weight is likely multifactorial. Obesity has been linked to diets characterized by high energy density but poor micronutrient quality, as well as to chronic low-grade inflammation, which increases hepcidin production, impairs intestinal iron absorption, and limits iron mobilization, thereby increasing the risk of iron deficiency and anemia (37).

Conversely, the inverse association observed in our study suggests that anemia may also reflect poorer dietary quality or inadequate nutritional intake, conditions generally associated with lower energy intake and consequently a lower likelihood of developing excess adiposity. Given the cross-sectional design of the present study, the direction of this association cannot be established, and the observed relationship should be interpreted as evidence of the complex interaction between dietary quality, inflammation, adiposity, and iron metabolism rather than a direct causal effect. The finding for non-flush sanitation may reflect broader household and socioeconomic conditions related to access to basic services and lifestyle patterns. Its consistency in the sensitivity analysis strengthens the observed pattern, although the underlying mechanisms require further investigation.

In contrast, in the primary analysis, urban residence was associated with lower odds of central obesity. Although the effect estimate remained similar in the sensitivity analysis accounting for within-school clustering, the association did not retain statistical significance. Comparable evidence among Peruvian school-aged children is scarce; however, a national analysis conducted in adult women reported a more rapid increase in obesity among those living in rural areas and in conditions of extreme poverty (22). Despite differences in the study populations, these findings suggest that place of residence may contribute to disparities in obesity risk across different stages of life. Non-piped water supply was also associated with higher odds of central obesity. This association may reflect broader socioeconomic and territorial conditions rather than a direct relationship between water access and adiposity, as access to basic services may coexist with differences in food access and lifestyle factors. However, this association also did not retain statistical significance in the sensitivity analysis, indicating that these contextual findings warrant cautious interpretation.

The lower odds of central obesity observed among children aged 11–13 years, which remained significant in the sensitivity analysis, may reflect age-related differences in growth and body composition during the transition from childhood to adolescence, a period characterized by substantial physiological and somatic changes (12, 13).

The inclusion of WHtR as a complementary indicator of adiposity strengthened these findings, as abdominal adiposity has been shown to be a better predictor of cardiometabolic risk than BMI alone in children (19, 38, 39). These findings support the potential utility of WHtR as a complementary indicator in nutritional screening of school-aged children. Historically, childhood obesity has been more prevalent in urban environments because of greater access to processed foods and sedentary lifestyles. However, growing evidence indicates that the nutrition transition is progressively extending to rural populations, reducing the traditional urban–rural differences in obesity prevalence (40).

### 4.3 Persistent anemia in school-aged children

Recent updates to the WHO hemoglobin thresholds have reduced the estimated prevalence of anemia among Peruvian children under five years of age, although substantial regional differences persist (41). Anemia affected 23.5% of schoolchildren in the present study indicating a substantial burden of anemia in the study population. Previous studies have reported even higher frequencies among children living in the Amazon (47.2%) (42) and Andean (48.8%) (43) regions, reflecting the marked geographical heterogeneity of anemia across the country. In contrast, studies conducted among children under five years of age have reported prevalences ranging from 5.2% to 66.7%, suggesting that the burden of anemia remains substantial throughout childhood despite ongoing national control strategies (44, 45).

A recent scoping review further highlighted the complexity of anemia in Peruvian children, identifying parasitic infections and iron deficiency as the most frequently reported etiological factors while emphasizing the lack of nationally representative data on the underlying causes of anemia (46). This multifactorial etiology reinforces the need to strengthen prevention and control strategies tailored to local epidemiological contexts. Regardless of its underlying cause, persistent anemia during childhood has important consequences for health and development. Iron deficiency has been associated with impaired cognitive development, reduced attention and learning capacity, diminished physical performance, and poorer quality of life, highlighting the importance of early detection and timely treatment (47).

Hemoglobin concentration also differed significantly according to age group and place of residence. The variation by residence is consistent with the marked geographical heterogeneity in childhood anemia previously reported in Peru (42, 44–46), whereas differences across age groups may partly reflect the physiological and somatic changes occurring throughout the school-age period (12). Our findings extend current evidence by showing that anemia affected nearly one in four schoolchildren in the study population, highlighting that anemia remains a relevant nutritional problem during the school years, even in a population characterized by a low prevalence of chronic undernutrition.

### 4.4 Double burden of malnutrition

The double burden of malnutrition (DBM) has been conceptualized using different approaches depending on the level of analysis and the objectives of each study. At the individual level, the classical definition refers to the coexistence of stunting and overweight or obesity (48, 49). However, epidemiological studies have also operationalized DBM using anemia as a proxy for micronutrient deficiency because hemoglobin concentration is routinely collected in population-based surveys and reflects one of the most common forms of hidden hunger (50, 51).

By applying an individual-level definition based on the coexistence of anemia and excess weight, the present study expands the epidemiological assessment of the DBM in Peru. This approach is particularly relevant to the study population, in which chronic undernutrition was uncommon whereas excess weight and anemia represented substantially more prevalent nutritional problems.

Using this operational definition, approximately one in ten schoolchildren presented the DBM, confirming that excess adiposity and anemia are not mutually exclusive conditions. Although, this prevalence is consistent with reports from other low- and middle-income countries (52). These findings demonstrate that the coexistence of anemia and excess weight is also present during the school years and are consistent with nutritional patterns described in populations undergoing the nutrition transition.

A recent systematic review reported differences in iron-related biomarkers between children with obesity and their normal-weight peers, including lower hemoglobin, serum iron, and transferrin saturation and higher ferritin and hepcidin concentrations (53).

The coexistence of anemia and excess weight has become increasingly recognized as a consequence of the ongoing nutrition transition in low- and middle-income countries. Previous analyses of Demographic and Health Survey (DHS) data from 16 countries reported a 43% increase in the prevalence of overweight among anemic children between the 1990s and early 2000s, suggesting that the overlap between these two forms of malnutrition is becoming progressively more common (51).

Regarding the factors associated with DBM, the multivariable analysis did not identify significant associations with sex, age group, or place of residence, despite the descriptively higher prevalence observed among rural schoolchildren. These findings were supported by the sensitivity analysis accounting for within-school clustering, in which no demographic factor was significantly associated with DBM. Similar findings have been reported in previous population-based studies, where individual-level DBM showed limited associations with demographic or socioeconomic characteristics (52). This suggests that demographic characteristics alone may not adequately capture the factors associated with individual-level DBM, which may involve dietary, biological, behavioral, and environmental factors related to both anemia and excess weight. Future studies incorporating dietary intake, micronutrient biomarkers, physical activity, and broader household and environmental characteristics are needed to better characterize the factors associated with DBM among school-aged children.

Overall, the coexistence of anemia and excess weight observed in this study highlights the presence of multiple forms of malnutrition among school-aged children, even in a population where traditional forms of undernutrition were uncommon. Considering anemia alongside indicators of excess adiposity may therefore provide a broader characterization of the nutritional profile of this population. These findings support the importance of integrated school health and nutrition strategies that address both anemia and excess weight rather than focusing on either condition in isolation.

### 4.5 School food environment

The descriptive assessment of the school food environment, based on the criteria established in the Peruvian national guidelines for healthy eating in educational institutions (Ministerial Resolution No. 195-2019/MINSA), showed widespread availability of industrially processed, energy-dense foods, including sugar-sweetened beverages, confectionery, industrially packaged cookies, and fried foods, whereas healthier options such as fresh fruits, vegetables, and healthy prepared foods were less frequently available. These findings characterize the food options available within the participating schools and should be interpreted descriptively. Because individual food purchases or dietary intake were not assessed, and the study was not designed to examine associations between school-level food availability and individual nutritional outcomes, no inference can be made regarding the contribution of the school food environment to excess weight or DBM.

The school food environment is increasingly recognized as an important environmental determinant of children's dietary behaviors and nutritional outcomes (54). Because children consume a substantial proportion of their daily food intake at school, the school setting represents a key opportunity to promote healthy eating habits and prevent childhood obesity (55). Furthermore, comprehensive school-based approaches have consistently demonstrated effectiveness in improving dietary behaviors, highlighting the importance of strengthening healthy school food environments and disseminating best practices across educational institutions (56).

In Peru, these findings are particularly relevant because Law No. 30021 (Law for the Promotion of Healthy Eating for Children and Adolescents) establishes that school kiosks should prioritize the sale of healthy foods and beverages while restricting products high in sugar, sodium, saturated fats, and trans fats. The widespread availability of these products in the participating schools suggests potential gaps in the implementation and monitoring of this legislation. Evidence indicates that well-designed school nutrition policies represent a feasible and effective strategy for improving children's dietary quality when appropriately implemented and monitored. Beyond improving food availability, international public health agencies recommend restricting children's exposure to the marketing of foods high in sugar, salt, and unhealthy fats. Recent WHO and UNICEF policy frameworks reinforce that comprehensive school nutrition policies combining healthier food environments with marketing regulations are essential to improve children's dietary behaviors (57–59).

Overall, our findings characterize a nutritional profile among school-aged children in Ica marked by a low prevalence of chronic undernutrition alongside a high burden of excess adiposity and anemia. The coexistence of anemia and excess weight highlights the importance of nutritional surveillance approaches that consider multiple forms of malnutrition rather than addressing these conditions separately. School-based health and nutrition strategies should therefore integrate anemia prevention with the promotion of healthy weight and healthy food environments. Further studies incorporating dietary intake, micronutrient biomarkers, physical activity, and broader environmental factors are needed to better understand the factors associated with individual-level DBM among school-aged children.

### 4.6 Limitations

This study has several limitations. First, its cross-sectional design precludes establishing temporal or causal relationships between the factors evaluated and nutritional outcomes. Second, schools were selected by convenience, which may limit the generalizability of the findings to all school-aged children in Ica or other regions of Peru. Third, hemoglobin concentration was used to identify anemia but does not allow determination of its underlying etiology, and individual dietary intake and physical activity were not assessed. Finally, the school food environment assessment was observational and reflects food availability rather than children's actual food purchases or consumption. In addition, participants were recruited from only six schools, limiting the precision of cluster-adjusted estimates. Although sensitivity analyses accounting for within-school clustering yielded effect estimates similar in magnitude to those from the primary models, inference from cluster-adjusted analyses should be interpreted cautiously because of the small number of school clusters.

## Data Availability

The datasets presented and analyzed in this study are available upon request from the corresponding authors.

## References

[1] Abarca-Gómez L Abdeen ZA Hamid ZA Abu-Rmeileh NM Acosta-Cazares B Acuin C . Worldwide trends in body-mass index, underweight, overweight, and obesity from 1975 to 2016: a pooled analysis of 2416 population-based measurement studies in 128·9 million children, adolescents, and adults. Lancet. (2017) 390:2627–42. doi: 10.1016/S0140-6736(17)32129-3 29029897 PMC5735219

[2] World Health Organization. Malnutrition in Children (2018). Available online at: https://www.who.int/data/nutrition/nlis/info/malnutrition-inchildren (Accessed May 20, 2026).

[3] World Health Organization. Obesity and Overweight (2025). Available online at: https://www.who.int/news-room/fact-sheets/dtail/obesity-and-overweight (Accessed May 20, 2026).

[4] Sommer A Twig G. The impact of childhood and adolescent obesity on cardiovascular risk in adulthood: a systematic review. Curr Diab Rep. (2018) 18:91. doi: 10.1007/s11892-018-1062-9 30167798

[5] Wang S Sun Q Zhai L Bai Y Wei W Jia L. The prevalence of depression and anxiety symptoms among overweight/obese and non-overweight/non-obese children/adolescents in China: a systematic review and meta-analysis. Int J Environ Res Public Health. (2019) 16:340. doi: 10.3390/ijerph16030340 30691096 PMC6388174

[6] Brambilla I Delle Cave F Guarracino C De Filippo M Votto M Licari A . Obesity and COVID-19 in children and adolescents: a double pandemic. Acta Biomed. (2022) 93:e2022195. doi: 10.23750/abm.v93iS3.13075 35666114 PMC9494180

[7] Alshwaiyat N Ahmad A Wan Hassan WMR. Al-jamal H. Association between obesity and iron deficiency (Review). Exp Ther Med. (2021) 22:1268. doi: 10.3892/etm.2021.10703 34594405 PMC8456489

[8] World Health Organization. The double burden of malnutrition: policy brief (2017). Available online at: https://www.who.int/publications/i/item/WHO-NMH-NHD-17.3 (Accessed May 20, 2026).

[9] Weffort VRS Lamounier JA. Hidden hunger - a narrative review. J Pediatr (Rio J). (2024) 100:S10–7. doi: 10.1016/j.jped.2023.08.009 37918810 PMC10960185

[10] Davis JN Oaks BM Engle-Stone R. The double burden of malnutrition: a systematic review of operational definitions. Curr Dev Nutr. (2020) 4:nzaa127. doi: 10.1093/cdn/nzaa127 32885132 PMC7456307

[11] Devine A Lawlis T. Nutrition and vulnerable groups. Nutrients. (2019) 11:1066. doi: 10.3390/nu11051066 31091644 PMC6566763

[12] Bundy DAP de Silva N Horton S Patton GC Schultz L Jamison DT. Child and adolescent health and development: realizing neglected potential. In: Disease Control Priorities, Third Edition (Volume 8): *Child and Adolescent Health and Development*. The World Bank (2017). p. 1–24. doi: 10.1596/978-1-4648-0423-6_ch1 30212145

[13] Martin FP Pinkney J Saavedra JM. Editorial: Nutrition and metabolism in school-age children. Front Nutr. (2022) 9:899126. doi: 10.3389/fnut.2022.899126 35571894 PMC9096345

[14] Instituto Nacional de Salud del Perú. Sala Situacional de Nutrición y Estudio del Sobrepeso y Obesidad (2023). Available online at: https://www.gob.pe/institucion/ins/informes-publicaciones/4408261-a-sobrepeso-y-obesidad-en-la-poblacion-peruana (Accessed September 16, 2026).

[15] Gobierno del Perú. Qali Warma realiza mesa de trabajo con ASPEC y Colegio de Nutricionistas del Perú para garantizar una adecuada alimentación escolar (2024). Available online at: https://www.gob.pe/institucion/wasimikuna/noticias/929902-qali-warma-realiza-mesa-de-trabajo-con-aspec-y-colegio-de-nutricionistas-del-peru-para-garantizar-una-adecuada-alimentacion-escolar (Accessed September 16, 2026).

[16] Instituto Nacional de Estadística e Informática. Ica: Compendio Estadístico 2025 (2025). Available online at: https://www.gob.pe/institucion/insm/informes-publicaciones/8613923-compendio-estadistico-2025 (Accessed May 20, 2026).

[17] Instituto Tecnológico de la Producción. Ica departamento: Economía (2018). Available online at: https://data-peru.itp.gob.pe/profile/geo/ica-1101#economia (Accessed June 15, 2026).

[18] World Health Organization. Growth reference data for 5–19 years (2007). Available online at: https://www.who.int/tools/growth-reference-data-for-5to19-years (Accessed June 15, 2026).

[19] Ashwell M Gibson S. Waist-to-height ratio as an indicator of “early health risk”: simpler and more predictive than using a “matrix” based on BMI and waist circumference. BMJ Open. (2016) 6:e010159. doi: 10.1136/bmjopen-2015-010159 26975935 PMC4800150

[20] World Health Organization. Guideline on haemoglobin cutoffs to define anaemia in individuals and populations (2024). Available online at: https://www.who.int/publications/i/item/9789240088542 (Accessed June 15, 2026). 38530913

[21] Rougeaux E Miranda JJ Fewtrell M Wells JCK. Maternal internal migration and child growth and nutritional health in Peru: an analysis of the demographic and health surveys from 1991 to 2017. BMC Public Health. (2022) 22:37. doi: 10.1186/s12889-021-12452-7 34991546 PMC8734138

[22] Santos MP Turner B Chaparro MP. The double burden of malnutrition in Peru: an update with a focus on social inequities. Am J Clin Nutr. (2021) 113:865–73. doi: 10.1093/ajcn/nqaa387 33564875

[23] Pomati M Mendoza-Quispe D Anza-Ramirez C Hernández-Vásquez A Carrillo Larco RM Fernandez G . Trends and patterns of the double burden of malnutrition (DBM) in Peru: a pooled analysis of 129,159 mother–child dyads. Int J Obes. (2021) 45:609–18. doi: 10.1038/s41366-020-00725-x 33402688 PMC7906898

[24] Mendoza-Quispe D Hernández-Vásquez A Miranda JJ Anza-Ramirez C Carrillo-Larco RM Pomati M . Urbanization in Peru is inversely associated with double burden of malnutrition: pooled analysis of 92,841 mother–child pairs. Obesity. (2021) 29:1363–74. doi: 10.1002/oby.23188 34148299 PMC8361670

[25] Popkin BM. Nutrition transition and the global diabetes epidemic. Curr Diab Rep. (2015) 15:64. doi: 10.1007/s11892-015-0631-4 26209940 PMC4942180

[26] Huicho L Huayanay-Espinoza CA Herrera-Perez E Segura ER Niño de. Guzman J, Rivera-Ch M, et al. Factors behind the success story of under-five stunting in Peru: a district ecological multilevel analysis. BMC Pediatr. (2017) 17:29. doi: 10.1186/s12887-017-0790-3 28103825 PMC5248498

[27] Huicho L Segura ER Huayanay-Espinoza CA de Guzman JN Restrepo-Méndez MC Tam Y . Child health and nutrition in Peru within an antipoverty political agenda: a Countdown to 2015 country case study. Lancet Glob Health. (2016) 4:e414–26. doi: 10.1016/S2214-109X(16)00085-1 27198845

[28] Hernández-Vásquez A Vargas-Fernández R Guerra Valencia J. Redefinición de la anemia infantil en el Perú: variaciones distritales tras la adopción de la guía OMS 2024. Acta Med Peru. (2025) 42:90–9. doi: 10.35663/amp.2025.422.3624

[29] Santos FDP Silva EAF Baêta CLV Campos FS Campos HO. Prevalence of childhood obesity in Brazil: a systematic review. J Trop Pediatr. (2023) 69:fmad017. doi: 10.1093/tropej/fmad017 36897068

[30] Poskitt EME. Childhood obesity in low- and middle-income countries. Paediatr Int Child Health. (2014) 34:239–49. doi: 10.1179/2046905514Y.0000000147 25203842

[31] Chis Ster I Alvarez-Mena P Zurita-Loma D Gualan M Rojeab-Bravo B Cooper PJ . Patterns of the triple burden of childhood malnutrition in Ecuador by age, sex, ethnicity, and poverty: a cross-sectional population survey. Lancet Reg Health Am. (2026) 60:101501. doi: 10.1016/j.lana.2026.101501 42256616 PMC13233759

[32] Hernández-Vásquez A Bendezú-Quispe G Santero M Azañedo D. Prevalence of childhood obesity by sex and regions in Peru, 2015. Rev Esp Salud Publica. (2016) 90:e1–10. PMID: 27619420. 27619420 PMC11587338

[33] Popkin BM Adair LS Ng SW. Global nutrition transition and the pandemic of obesity in developing countries. Nutr Rev. (2012) 70:3–21. doi: 10.1111/j.1753-4887.2011.00456.x 22221213 PMC3257829

[34] Twig G Yaniv G Levine H Leiba A Goldberger N Derazne E. et al. Body-mass index in 23 Million adolescents and cardiovascular death in adulthood. N Engl J Med. (2016) 374:2430–40. doi: 10.1056/NEJMoa1503840 27074389

[35] Biro FM Wien M. Childhood obesity and adult morbidities. Am J Clin Nutr. (2010) 91:1499S−505S. doi: 10.3945/ajcn.2010.28701B 20335542 PMC2854915

[36] Llewellyn A Simmonds M Owen CG Woolacott N. Childhood obesity as a predictor of morbidity in adulthood: a systematic review and meta-analysis. Obes Rev. (2016) 17:56–67. doi: 10.1111/obr.12316 26440472

[37] Teng I Tseng S Aulia B Shih C Bai C Chang J. Can diet-induced weight loss improve iron homoeostasis in patients with obesity: a systematic review and meta-analysis. Obes Rev. (2020) 21:e13080. doi: 10.1111/obr.13080 32677171

[38] Lo K Wong M Khalechelvam P Tam W. Waist-to-height ratio, body mass index and waist circumference for screening paediatric cardio-metabolic risk factors: a meta-analysis. Obes Rev. (2016) 17:1258–75. doi: 10.1111/obr.12456 27452904

[39] Powell-Wiley TM Poirier P Burke LE Després JP Gordon-Larsen P Lavie CJ . Obesity and cardiovascular disease: a scientific statement from the American Heart Association. Circulation. (2021) 143:e984–e1010. doi: 10.1161/CIR.0000000000000973 33882682 PMC8493650

[40] Paciorek CJ Stevens GA Finucane MM Ezzati M. Children's height and weight in rural and urban populations in low-income and middle-income countries: a systematic analysis of population-representative data. Lancet Glob Health. (2013) 1:e300–9. doi: 10.1016/S2214-109X(13)70109-8 25104494 PMC4547325

[41] Campos-Sánchez M Cordero-Muñoz L Velásquez-Hurtado E Baiocchi-Ureta N Miranda-Cuadros M Sánchez-Griñán MI . New world health organization guideline on anemia cut-off points: implications for children aged 6-35 months in Peru. Rev Peru Med Exp Salud Publica. (2025) 42:115–25. doi: 10.17843/rpmesp.2025.422.14028 40900478 PMC12377884

[42] Segoviano-Lorenzo M. del C, Trigo-Esteban E, Gyorkos TW, St-Denis K, Guzmán FMD, Casapía-Morales M. Prevalence of malnutrition, anemia, and soil-transmitted helminthiasis in preschool-age children living in peri-urban populations in the Peruvian Amazon. Cad Saude Publica. (2022) 38:e00248221. doi: 10.1590/0102-311xen248221 36449752

[43] Vásquez-Velásquez C Choque-Quispe BM Suchdev PS Rees CA Tapia V Ko YA . Changes in anemia prevalence and the proportion of anemia associated with iron deficiency or inflammation in young children residing in Puno, Peru: Analysis using new World Health Organization guidelines for defining anemia. PLoS One. (2026) 21:e0342255. doi: 10.1371/journal.pone.0342255 41719324 PMC12923139

[44] Cerda-Hernandez J Sikov A Vidal-Valenzuela LY. Análisis espacial de la anemia infantil en Perú 2022: construcción de mapas a nivel distrital para políticas públicas. Salud Publica Mex. (2024) 66:236–44. doi: 10.21149/15206 39977073

[45] Choque-Quispe BM Alarcón-Yaquetto DE Paredes-Ugarte W Zaira A Ochoa A Gonzales GF. Is the prevalence of anemia in children living at high altitudes real? An observational study in Peru. Ann N Y Acad Sci. (2020) 1473:35–47. doi: 10.1111/nyas.14356 32374436

[46] Lobo K Vásquez-Velásquez C Suchdev PS Young MF Rogers H Cyrus E . Anemia crisis among children in Peru: a scoping review. Rev Panam Salud Publica. (2026) 50:1. doi: 10.26633/RPSP.2026.58 42453945 PMC13367210

[47] Powers JM Heeney MM Hord J Lehmann CU Abrams SA Buchanan GR . Prevention, screening, diagnosis, and treatment of iron deficiency and iron deficiency anemia in infants, children, and adolescents: clinical report. Pediatrics. (2026) 158:e2026077414. doi: 10.1542/peds.2026-077414 42324084

[48] Zhang Y Huang X Yang Y Liu X Yang C Wang A . Double burden of malnutrition among children under 5 in poor areas of China. PLoS One. (2018) 13:e0204142. doi: 10.1371/journal.pone.0204142 30222775 PMC6141094

[49] Popkin BM Corvalan C Grummer-Strawn LM. Dynamics of the double burden of malnutrition and the changing nutrition reality. Lancet. (2020) 395:65–74. doi: 10.1016/S0140-6736(19)32497-3 31852602 PMC7179702

[50] Rivera JA Pedraza LS Martorell R Gil A. Introduction to the double burden of undernutrition and excess weight in Latin America. Am J Clin Nutr. (2014) 100:1613S−6S. doi: 10.3945/ajcn.114.084806 25411302

[51] Jones AD Acharya Y Galway LP. Urbanicity gradients are associated with the household- and individual-level double burden of malnutrition in Sub-Saharan Africa. J Nutr. (2016) 146:1257–67. doi: 10.3945/jn.115.226654 27170726

[52] Engle-Stone R Guo J Ismaily S Addo OY Ahmed T Oaks B . Intraindividual double burden of overweight and micronutrient deficiencies or anemia among preschool children. Am J Clin Nutr. (2020) 112:478S−87S. doi: 10.1093/ajcn/nqaa101 32743648 PMC7396269

[53] Berton PF Gambero A. Hepcidin and inflammation associated with iron deficiency in childhood obesity - a systematic review. J Pediatr. (2024) 100:124–31. doi: 10.1016/j.jped.2023.06.002 37541648 PMC10943301

[54] Farmer KO Beukes RA Sowden M du Plessis LM. The impact of food and nonalcoholic beverage sponsorship and marketing in the school environment on the food choices of school-going children and adolescents: a scoping review, 2011–2023. Obes Rev. (2025) 26:e13923. doi: 10.1111/obr.13923 40156044 PMC12246883

[55] Welker E Lott M Story M. The school food environment and obesity prevention: progress over the last decade. Curr Obes Rep. (2016) 5:145–55. doi: 10.1007/s13679-016-0204-0 27066793

[56] Langford R Bonell CP Jones HE Pouliou T Murphy SM Waters E . The WHO Health Promoting School framework for improving the health and well-being of students and their academic achievement. Cochrane Database Syst Rev. (2014) 2014:CD008958. doi: 10.1002/14651858.CD008958.pub2 24737131 PMC11214127

[57] World Health Organization. Policies to protect children from the harmful impact of food marketing: WHO guideline (2023). Available online at: https://www.who.int/publications/i/item/9789240075412 (Accessed June 15, 2026). 37440677

[58] World Health Organization. Taking action to protect children from the harmful impact of food marketing: a child rights-based approach (2023). Available online at: https://www.who.int/publications/i/item/9789240047518 (Accessed June 15, 2026).

[59] Boachie MK Goldstein S Kruger P Ng SW Hofman KJ Thsehla E. Beverage industry's advertising expenditures and airtimes in South Africa from 2013 to 2019 target children and families. J Public Health Res. (2023) 12:22799036231168207. doi: 10.1177/22799036231168207 37122639 PMC10134126

