# Supplementary material for: The double burden of anemia and excess weight among schoolchildren in Southern Peru: prevalence, associated factors, and the school food environment
Source: Front Nutr. 2026 Oct 28;13:1965478. doi: 10.3389/fnut.2026.1965478 (PMC13752043; doi:10.3389/fnut.2026.1965478)
Supplement: Supplementary file 1 [file Table_1.docx]

Supplementary Material

Supplementary Table S1. Sensitivity analysis accounting for clustering of schoolchildren within schools using generalized estimating equations

| **Outcome** | **Variable** | **aOR** | **95% CI** | **P-value** |
| --- | --- | --- | --- | --- |
| Double burden of malnutrition | Sex: Male | 1.32 | [0.95–1.82] | 0.098 |
|  | Age: 8–10 years | 0.80 | [0.50–1.29] | 0.365 |
|  | Age: 11–13 years | 0.58 | [0.32–1.04] | 0.069 |
|  | Place of residence: Urban | 0.66 | [0.28–1.60] | 0.360 |
| Excess weight | Sex: Male | **1.30** | [**1.02–1.65**] | **0.035** |
|  | Age: 8–10 years | 0.88 | [0.60–1.30] | 0.524 |
|  | Age: 11–13 years | 0.87 | [0.40–1.86] | 0.714 |
|  | Place of residence: Urban | 1.07 | [0.86–1.34] | 0.536 |
|  | Anemia | **0.63** | [**0.41–0.96**] | **0.032** |
|  | Cement floor | 1.41 | [0.66–3.02] | 0.379 |
|  | Non-piped water supply | 1.36 | [0.81–2.28] | 0.240 |
|  | Non-flush sanitation | **2.01** | [**1.20–3.36**] | **0.008** |
| Central obesity | Sex: Male | 1.23 | [0.87–1.74] | 0.246 |
|  | Age: 8–10 years | 0.70 | [0.41–1.19] | 0.188 |
|  | Age: 11–13 years | **0.56** | [**0.35–0.88**] | **0.013** |
|  | Place of residence: Urban | 0.63 | [0.38–1.02] | 0.061 |
|  | Anemia | **0.71** | [**0.51–0.98**] | **0.037** |
|  | Non-piped water supply | 1.68 | [0.78–3.65] | 0.188 |

aOR, adjusted odds ratio; CI, confidence interval. Models were estimated using generalized estimating equations (GEE) with a binomial distribution, logit link, school as the clustering variable, and an exchangeable working correlation structure. A Mancl–DeRouen small-sample correction was applied to the sandwich variance estimator because only six schools were included. Reference categories were female sex, age 5–7 years, and rural residence. Bold values indicate *p* < 0.05.
